# Fruit Pod Extracts as a Source of Nutraceuticals and Pharmaceuticals

**DOI:** 10.3390/molecules171011931

**Published:** 2012-10-10

**Authors:** Azila Abdul Karim, Azrina Azlan

**Affiliations:** 1 Cocoa Innovation & Technology Centre, Malaysian Cocoa Board, PT12621, Nilai Industrial Area, 71800 Nilai, Negeri Sembilan, Malaysia; Email: aziela@koko.gov.my; 2 Department of Nutrition and Dietetics, Faculty of Medicine and Health Sciences, Universiti Putra Malaysia, 43400 UPM Serdang, Selangor, Malaysia; 3 Laboratory of Halal Science Research, Halal Products Research Institute, Universiti Putra Malaysia, 43400 UPM Serdang, Selangor, Malaysia

**Keywords:** fruit pods, bioactive, nutraceutical, pharmaceutical

## Abstract

Fruit pods contain various beneficial compounds that have biological activities and can be used as a source of pharmaceutical and nutraceutical products. Although pods or pericarps are usually discarded when consuming the edible parts of fruits, they contain some compounds that exhibit biological activities after extraction. Most fruit pods included in this review contain polyphenolic components that can promote antioxidant effects on human health. Additionally, anti-inflammatory, antibacterial, antifungal and chemopreventive effects are associated with these fruit pod extracts. Besides polyphenolics, other compounds such as xanthones, carotenoids and saponins also exhibit health effects and can be potential sources of nutraceutical and pharmaceutical components. In this review, information on fruit pods or pericarp of *Garcinia mangostana*, *Ceratonia siliqua*, *Moringa oleifera*, *Acacia nilotica*, *Sapindus rarak* and *Prosopis cineraria* is presented and discussed with regard to their biological activity of the major compounds existing in them. The fruit pods of other ethno- botanical plants have also been reviewed. It can be concluded that although fruit pods are considered as being of no practical use and are often being thrown away, they nevertheless contain compounds that might be useful sources of nutraceutical and other pharmaceutical components.

## 1. Introduction

A nutraceutical is defined as any substance that is food or a part of food that provides medical or health benefits, for the prevention and treatment of diseases [1]. Nutraceuticals include a broad range of categories such as dietary supplements, functional foods and herbal products [2]. The active compounds or phytochemicals in plants, especially fruits, have been associated with numerous health benefits [3] and are used as ingredients in many nutraceutical and pharmaceutical products today. Radhika *et al*. [2] listed some sources of active ingredients from plants being used in manufacture of nutraceuticals. There are at least fourteen classes of secondary metabolites (chemical compounds) from fruits and vegetables that exert biological activities and can potentially be used to promote human health. These include alkaloids, amines, cyanogenic glycosides, diterpenes, flavonoids, glucosinolates, monoterpenes, non-protein amino acids, phenylpropanes, polyacetylenes, polyketides, sesquiterpenes, tetraterpenes, triterpenes, saponins and steroids [4]. Research by Mukherjee *et al.* [5] highlighted some chemical compounds from from various parts of plants that exhibit potential antioxidant activities, including madecossoside, asiaticoside, catechin, epicatechin, 4-hydroxycinnamic acid, esculetin, curcumin, xanthorrhizol, anthocyanins, diosgenin, gallic acid, ginsenoside, β-carotene, ginsenoside and cyanidin-3-glucoside. However, plant extracts can be toxic and contain excessive lethal constituents such as aristolochic acids, pyrrolizidone alkaloids, benzophenanthrine alkaloids, viscotoxins, saponins, diterpenes, cyanogenetic glycosides and furanocoumarins [6]. These compounds can affect human health since nutraceutical products, unlike pharmaceutical products, are not as well regulated and are commonly consumed without supervision or medical guidance. On the other hand, phenolic compounds from a variety of fruits such as catechin, anthocyanins, quercetin, kaempherol, resvasterol, curcuminoids, genistein, apigenin, carotenoids, carnosic acid, caffeic acid and ferulic acid are known to possess antioxidant activities and a sun-protective effect against UV light-induced damage [7]. Catechin, for example, has potent biological activity in cancer prevention, and has antioxidant, cardiovascular protection and hepatoprotective properties [8].

Pods are usually discarded when consuming fruits. The pod is the outer layer of some fruits which is hard in texture and is sometimes too bitter or astringent to be eaten raw, as in the case of mangosteen and cocoa. Pods are also called pericarps or rinds that surround the seeds [9]. The pericarp consists of three main parts, namely the epicarp or exocarp, mesocarp and endocarp. The outermost part, the epicarp, is usually called the skin or peel of the fruits. The middle layer, mesocarp, can be edible in some fruits such as mango, or fibrous like in palm oil fruit. Finally, the endocarp encloses the seeds. It occurs in various forms, such as the hard shell of coconuts or the soft shell of cocoa [10]. In between the mesocarp and endocarp, there is also a part called the aril or placenta of the seed that can be consumed. This part is usually white in color and juicy as an attractant to animals in order for the plant to grow diversely [11].

Representing the outer part of the fruits, the pericarp comes in various colors and changes during ripening depending on the types of fruits. For example, cocoa pods when ripened turn yellow from either red maroon or green. As another example, ripe mangosteens turn dark purple from green. Some pods turn brown, black or dark brown. The color results from pigments and phytochemicals such as chlorophyll, carotenoids and phenolics [12]. Red fruits are associated with compounds like lycopene, ellagic acid, quercetin and hesperidin, while orange and yellow fruits are usually linked to β-carotene, zeaxanthin, flavonoids and vitamin C [13]. Chlorophyll, lutein, zeaxanthin and β-carotene are related to green fruits [14]. Blue and purple colors of fruits are often applied to resveratrol, quercetin and ellagic acid [15]. Finally, white colour is associated with the presence of β-glucans and lignans [16]. Although tristimulus colorimeters can be used to measure the visual appearance of fruit pericarp [17], identifying the profiles of the pericarp content should be carried out by analytical instrumentation to confirm the exact active compounds that exist in it.

This review focuses on bioactive compounds or phytochemicals of fruit pods with beneficial health effects that have potential pharmaceutical and nutraceutical applications. This review provides researchers with useful knowledge and guidance in future experimental work on developing pharmaceutical and nutraceutical products from this part of fruits. The available literature was searched using Google Scholar, Science Direct and Springer Link for scientific publications published during the period 1992–May 2012 describing beneficial aspects of bioactive components from fruit pods. In fact, certain fruits like mangosteen, carob, *Acacia nilotica* and *Moringa oleifera* and their pods have been extensively studied. Keywords used for the search were bioactive compounds, fruit pods, nutraceutical, pharmaceutical and plant names. This review focuses on the beneficial effects of bioactive compounds from fruit pods through the review by available *in-vitro*, pre-clinical and/or human trial studies in the related literature.

## 2. Garcinia mangostana

*G. mangostana* is also known as mangosteen or *manggis* in Malaysia. Although this plant grows well in tropical areas of the World, including Malaysia, Thailand and Indonesia, the nutraceutical products of mangosteen have been patented by Garrity *et al.* [18] from the USA and successfully marketed worldwide. Two major groups of phytochemicals in the mangosteen pericarp are the xanthones and phenolics. The antioxidant properties of *G.mangostana* is related to the presence of high levels of phenolic compounds (tannins) in its methanol, and ethanol extracts [19]. The phenolic compounds in mangosteen, including afzelechin, epiafzelechin, catechin, epicatechin, gallocatechin and epigallocatechin, can proiduce oxygen radical scavenging capacities as high as 1.7 × 10^4^ μmol TE/g, which is greater than those of grape seed and pine bark [20]. The quantity of phenolic compounds and antioxidant activity in the mangosteen pericarp is ten times and 20 times greater, respectively, than in the white edible parts. 

Identification of mangosteen pericarp components, carried out by Asai *et al.* and Jung *et al.* [21,22], revealed that the xanthone group includes α-, β-, and γ-mangostins, gartanin, garcinone E, garcinone D, tovophyllin, mangostinone, smeathxanthone, 1-isomangostin, eudraxanthone G, 1,5-dihydroxy-2-(3-methylbut-2-enyl)-3-methoxy- and 1,7-dihydroxy-2-(3-methylbut-2-enyl)-3-methoxyxanthone. A high content of xanthones has been detected in the pericarp when compared with the white aril part of the fruits, with seven main components identified as the fingerprint of mangosteen extract (1,7-dihydroxy-3-methoxy-2-(3-methylbut-2-enyl)xanthone, γ-mangostin, 8-deosygartanin, 1,3,7-trihydroxy-2,8-di(3-methylbut-2-enyl)xanthone, gartanin, α-mangostin and garcinone E) [23]. The xanthones also exhibited strong antioxidant properties. The extract of mangosteen rind in dichloromethane revealed that the xanthone compounds are also present in high concentrations; especially α-mangostin that exhibited the strongest activity against bacteria that induce acne, including *Propionibacterium acnes*, *Staphylococcus epidermidis* [24] and oral candidiasis, *Candida albicans* [25]. The compound α-mangostin isolated from mangosteen extract (1.0 μg/mL) also showed the most effective inhibitory effect against a preneoplastic lesion (leading to breast cancer) in a mouse mammary organ study [22].

Medicinal properties of *G. mangostana* have been summarized by Chaverri *et al.* [26] and show that the extract of this pericarp has antioxidant, antitumor, anti-inflammatory, antiallergy and antimalarial, besides anti-bacterial/viral properties. In addition, studies on the effect of mangosteen extract on obesity genes have also shown positive results [27,28,29]. The extract also reduced cholesterol level in rats [30]. The crude methanolic extract of mangosteen strongly inhibited human breast cancer cell profileration at ED_50_ of 9.25 μg/mL [31] and colon cancer cells [32]. In another study, crude methanolic extract of mangosteen also inhibited quinone reductase activity [33]. Nakatani *et al.* [34] studied the anti-allergy and anti-inflammatory effects of mangosteen pericarp extract in comparison with that of a traditional plant extract of *Rubus suavissimus* in Japan using *in vitro* studies. Mangosteen ethanol extract (40%) inhibited histamine release and prostaglandin synthesis with greater effect. The extract also inhibited the synthesis of cyclooxygenase [35], a rate-limiting enzyme, functionally important in fluid and electrolyte homeostasis, gastric acid secretion and platelet aggregation. Ethanolic (50%) extract of *G.mangostana* has been shown to exhibit neuroprotective activity *in vitro* [36]. Human subjects who received mangosteen dietary supplements for one month also showed significantly enhanced immune responses [37]. On the other hand, polysaccharide from mangosteen pericarp extracted using hot water and precipitated with ethanol stimulated phagocytic cells and demolished intracellular bacteria, namely *Salmonella enteritidis*. The extract contained D-gallacturonic acid, L-rhamnose and D-galactose [38]. A different species of mangosteen, *Garcinia cochinchinensis*, also exhibited strong anticancer activity following the presence of the compound guttiferone. The extract of *G.cochinchinensis* pericarp was higher in xanthone as well as trimethyl citrate [39].

Most of pharmaceutical and nutraceutical properties of *G. mangostana* pod extract in the scientific papers were studied using *in-vitro* methods where the effects varied between cells and species depending on host metabolism and bioavailability of the extracted compounds. For example, the effective concentration of extracts to cancer cells of human was ten times higher than needed for mouse to inhibit proliferation of cells *in-vitro*. Therefore, human clinical trials are needed due to the different *in-vitro* potency. Meanwhile, the extraction method such as type and percentage of solvent used can affect the beneficial response such as a hot water prepared extract is lacking in glucose, as previously shown [38], thus requiring detailed identification of the compounds present the extract.

## 3. Ceratonia siliqua

*C. siliqua* is a plant of the Mediteranean regions. Also known as carob, the crude extract of the plant pod exhibited antioxidant properties higher than certain known polyphenols such as catechin, quercetin and gallic acid alone [40,41], due to the presence of carotenoids such as lutein, lycopene, α-carotene and β-carotene. Compound identification using High Performance Liquid Chromatography (HPLC) showed that carob pods contain flavonoids of quercetin glycosides, catechin and epicatechin gallate, polyphenols of gallic acid and ellagic acid, and anthocyanins such as proanthocyanidins and ellagitannins [42], as well as epigallocatechin gallate [43]. 

Avallone *et al.* [44] revealed that extract of carob pod can be used as a natural product with anxiolytic-sedative effects and act as a chemopreventive agent. The presence of gallic acid, epigallocatechin-3-gallate and epicatechin-3-gallate in carob pod can exert antiproliferative effects *in-vitro* [45]. Proliferative effects are related to cancerous cells that grow and increase rapidly. The patented aqueous extract of *C. siliqua* pod revealed the antioxidant activity of the extract along with potential antitumor activity [43]. The extract has equivalent antioxidant level to tea, but without the stimulant effects of caffeine and theophyline. The extract was prepared using hot distilled water for 15 minutes, then filtered and evaporated to dryness. The polyphenols content of the extract was 1.36 mg/g of pod powder. The pod extract at a concentration of 80 μg/mL was shown to effectively inhibit proliferation in liver tumor cells after 48 hours of treatment *in-vitro*. The extract of carob pod also has an antidepressant effects as exhibited in a study by Agrawal *et al.* on mice using tail suspension and forced swim tests [46]. The anti-diarrhea effect of carob pod dietary fiber, as patented by Mark *et al.* was observed in tube-fed patients [47]. Dietary fiber of carob pod also proved to reduce total cholesterol and LDL-c levels significantly after six weeks of consumption before breakfast in 49 volunteers with mild to moderate cholesterol levels [48].

Generally, pod extracts of *C. siliqua* were shown to posesses health benefits for humans. However, the *in vitro* antiproliferative and other beneficial effects need to be confirmed and extensively studied to evaluate its effectiveness *in-vivo*. The high antioxidant level of *C. siliqua* pod extract suggesting its potential development as a nutraceutical or pharmaceutical product.

## 4. Moringa oleifera

*M. oleifera* is a semi-arid plant [49], which can be found in tropical and subtropical climates. The plant, which is also known as the horseradish tree, can be found in India, Thailand, Africa and Indonesia. Research has shown that biologically active components present in this plant contributing to its health benefits are simple sugars and compounds called glucosinolates and isothiocyanates, including 4-(4′-*O*-acetyl-α-L-rhamnopyranosyloxy)benzyl isothiocyanate, niazimicin, pterygospermin, benzyl isothiocyanate and 4-(α-L-rhamnopyranosyloxy)benzyl glucosinolate, as well as carotenoids [50]. Two other compounds, niaziridin and niazirin, were also detected in *M. oleifera* pod by reverse phase HPLC [51]. 

Reportedly, *M. oleifera* pod has antioxidant activity [52], which is due to the presence of carotenoid compounds. The pod contains high amounts of bio-enhancers in comparison with the bark. It can be used to reduce cholesterol and glucose in blood with the safe intake level determined at ≤1,000 mg/kg body weight [53]. The ethanolic pod extract also showed hypotensive activity at a 30 mg/kg dose [54]. According to Jakansul *et al.* [55], the hypertensive effect was due to the thiocarbamate glycoside extracted from the pod. Intake of pod powder of *M. oleifera* was also shown to cause significant reduction in total cholesterol and total lipid levels of high lipid and high cholesterol diet-induced rabbits [56].

Antiurolithiatic activity was also exhibited by the extract of *Moringa oleifera* pods as studied by Vijayalakshmi *et al.* [57]. The extraction was carried out using boiling water for six hours and evaporation to dryness. Urolithiasis-induced albino rats were treated with the extract for one month. In comparison with the control group, rats fed with the plant extract at 400 mg/kg body weight showed significant reduction in stone weight. 

A significant anti-inflammatory action was revealed by Rakesh *et al.* [58] in a study of the effects of *M. oleifera* ethanolic extract on carrageenan-induced paw edema in albino mice. The study was carried out to compare the relative effect of pod extract to diclofenac sodium, where the required dose of pod extract was 1,000 mg/kg. Methanolic extract of fresh *M. oleifera* fruits also exhibited an anti-inflammatory effect *in-vitro* as observed by Cheenpracha *et al.* and Muangnoi *et al.* [59,60]. 

Aqueous ethanolic extract of *M. oleifera* also has a hepatoprotective effect, which was exhibited in albino mice with induced hepatocarcinogeniticity [61]. The effectiveness of pod extract was also apparent in repairing liver damaged by carbon tetrachloride in albino mice at 750 mg/kg [62]. Moreover, a niaziridin-rich extract fraction of *M. oleifera* pods enhanced the bioactivity of several antibiotics (rifampicin, tetra cycline and ampicillin) against bacteria and facilitated drug absorption through the gastrointestinal membrane [63].

The above studies indicate the beneficial effects on health by *M. oleifera* pod extracts. Although most of the effects were observed in animal models, these benefits may be extended to humans in the form of dietary supplements in the future.

## 5. Acacia nilotica

Planted in the Arabian Peninsula, Pakistan, India and Burma, *Acacia nilotica* is also known as the gum arabic tree. The pod of this Egyptian medicinal plant contains gallocatechin 5-*O*-gallate, methyl gallate, gallic acid, catechin, catechin 5-*O*-gallate, 1-*O*-galloyl-β-D-glucose, 1-6-di-*O*-galloyl-β-D-glucose and digallic acid. Its green pod is high in gallic acid, elagic acid, ferulic and epicatechin [8]. 

Formerly, the plant extract was used as a tanning [64] and dying agent. The plant itself was very useul since it provided water and insect-resistant wood, fodder for livestock and folk medicines. The pods were used to treat fever, diarrhea, diabetes, sore gums and skin diseases. Studies indicate that the pod extract of *A. nilotica* exhibits antioxidant, chemopreventive, antidiabetic, hypolipidemic and antiplasmodial activities [64,65]. The extraction was carried out by soaking of 44.2 g *A. nilotica* pod overnight in 500 mL of methanol three times. After filtration and solvent removal, the crude extract was fractionated using the solvents *n*-hexane, methanol, ethyl acetate, buthanol and water [8]. The aqueous methanolic extract of *Acacia nilotica* pod also caused significant reduction of blood glucose, plasma total cholestrol, triglycerides and low-density lipid (LDL) levels in alloxan-induced diabetic rabbits at 400 mg/kg body weight in a one-month treatment [66]. The ethyl acetate fraction of the pod exhibited antidiarrheal activity in albino rats at 400 mg/kg body weight [67]. Freeze dried extract of *A. nilotica* pods showed bactericidal activity against an extended spectrum of β-lactamase (ESBL) and methicillin-resistant *Staphylococcus aureus* (MRSA), as studied previously [68]. Another species of acacia, *Acacia auiculiformis*, has abundant amounts of acaciaside (tripernoid) in its pericarp that exhibits antifilarial activity [69].

Nutraceutical products could possibly be produced by using *A. nilotica* pod extract as a source of dietary fibre. Even though effective antidiarrheal effects have been determined in animals models, its efficacy in humans also need to be addressed in future studies.

## 6. Sapindus rarak

*Sapindus rarak* is the soapberry plant, known in Indonesia as *lerai* or *kelerak*. Traditionally, the pericarp was used for skin disorders. Four triterpene saponins have been identified in *S. rarak* pericarp, including rarasaponins (I, II, and III), raraoside, along with 13 other known saponins and four known acyclic sesquiterpene glycosides. A study revealed that the methanolic extract of *Sapindus rarak* pericarp contains saponins that inhibited pancreatic lipase and reduced lipid digestion *in-vitro* with an IC_50_ of 614 µg/mL [70]*.* The antiobesity effects of this plant were more strongly exhibited by raraoside and rarasaponin in comparison with the saponin compounds. A study on the effects of the methanolic extract of *Sapindus rarak* on plasma trigylceride elevation was conducted in mice treated with olive oil [71]. The results indicated that elevation of triglycerides was inhibited at a dose of 200 mg/kg by the saponin constituents, including hederagenin. The extract also exhibited inhibitory effects on tumor necrosis at a concentration of 30–100 µM, as reported by Morikawa *et al.* [72].

*S. rarak* has potential to be used in the formulation of nutraceutical products with weight loss effects due to the presence of bioactive compounds that inhibit lipid digestion. However, the effective dose of extract must be determined prior to its incorporation into products due to its high content of saponins. 

## 7. Prosopis cineraria

*P. cineraria* or Khejri is also known as the king of the desert. The plant grows well in Western and Southern Asia, including Afghanistan, Iran, India, Oman, Pakistan and Saudi Arabia. When the boiling water extract of its pods is fractionated using methanol and trichloromethane, it results in the isolation of compounds such as 3-benzyl-2-hydroxy-urs-12-en-28-oic acid and maslinic acid-3-glucoside (triterpernoids); linoleic acid (fatty acid); prosophylline (piperidine alkaloid); 5,5′-oxybis-1,2-benzanediol; 3,4,5-trihydroxycinnamic acid 2-hydroxyethyl ester; and 5,3′,4′-trihydroxyflavanone 7-glycoside (polyphenols). *In vitro* bioassays carried out by Liu *et al*. [73] using these compounds and crude extract have shown positive results supporting its health benefits in preventing a wide range of illnesses including protein and mineral deficiencies. High antioxidant activity has also been exhibited by the methanolic extract of its pods, which also have indicated antimicrobial activity against *Candida albicans* [74]. There is also empirical proof that *P. cineraria* can exhibit estrogenic activity *in vitro* [75].

Although most of the biological activities showed in studies were proven in animals, there are potential benefits of *P. cineraria* pod extract as an antioxidant. However, the toxicity effects of the extracts need to be determined as the extract contains piperidine alkaloids which might exert certain adverse effects when consumed, as mentioned by Bahorun *et al.* [6].

## 8. Other Ethnobotanicals

There are several other plant pods which are used in traditional medicines for health and medicinal (ethnobotanical) purposes. However, most of the related publications focus on the biological activity of the pod crude extracts without any detailed identification of the extract itself. Ethanol extract of unripe *Bauhinia purpurea* exhibited an anti-obesity effect in cholesterol and high fat diet (CHFD)-induced hyperlipidemic rats [76]. As it was observed in this study, feeding the rats with CHFD together with unripe pods at 300 mg/kg/day lowered the body weight increase by 7.4% in comparison with hyperlipidemic rats (13.11%) in the control group. The crude extract of unripe *B. purpurea* pods was reported to contain carbohydrates, proteins, alkaloids, flavonoids, triterpenes, glycosides and steroids. 

*Parkia speciosa* or stinky bean (*petai* in Malaysia) has also exhibited antioxidant properties due to its high total phenolic and high flavonoid contents [77]. Oral administration of chloroform extracts of *P. speciosa* pod to diabetic-induced rats resulted in a significant reduction of blood glucose levels which suggests the potential use of the extract as an oral hypoglyceamic agent [78]. The methanolic extract of the *Parkia speciosa* pod also exhibited a significant antiangiogenic effect [79]. The ethanol extract of *Cassia occidentalis* pod showed antifungal activity towards *Aspergillus clavatus* at 100 μg/mL, which was equal to the concentration required using commercial drugs such as nystatin and griseofulvin. Although a higher minimum inhibitory concentration (MIC) value was needed to inhibit *Candida albicans* and *Aspergillus niger* (125 and 500 μg/mL, respectively), the inhibitory effect of the pod extract was comparable to the said drugs [80]. 

The extract of *Albizia julibrissin Durazz* pod exhibits antioxidant activity and a strong inhibition against *E. coli*, *S. aureus*, *B. megaterium*, *B. subtilis* and *S. typhi* [81]. However, a report [82] indicated that methanolic extract of *Albizza lebbeck* pod decreased the fertility of male albino rats significantly, without stating whether the effect was reversible or not. Nearly, 45% of the pod compounds are aromatic. *n*-Hexane extract of *Samanea saman* pods contains phytochemicals such as cyanidin, catechin, epicachin, anthocyanin monoglycones, delphinidin and malidin. Potent antibacterial properties, including against *Candida albicans*, have been exhibited by the ethyl acetate fraction of *Samanea saman* at 10,000 ppm [83]. Moderate antibacterial activity against *Bacillus subtilis* and *Pseudominas aeruginosa* was exhibited by the water extract of *Cassia fistula* pod [84]. 

Fruit pericarp of *Emblica officinalis* (used in Ayurveda treatment) contains hydrolysable tannins such as emblicanin, punigluconin and pedunculagin with significant antioxidant properties [85]. Extracts of *Catalpa bignonoides* pods exhibit anti-inflammatory and antinociceptive effects due to the presence of saponin, sterol or phenol compounds in the pods [86]. These properties are also exhibited by *Tecoma sambucifolia* [87]. Anti-inflammatory activity has also been observed in polysacharide extract of *Caesalpinia ferrea* pods [88]. Beneficial effects of *Astragalus hamosus* pod extract on edema-induced rats has also been reported by Hakim *et al.* [89], who observed a significant reduction in the size of rats’ hind paws 3 hours after injection. The aqueous and alcoholic extracts of the pod exhibit a similar significant effect. Methanol extract of *Caesalpinia pulcherrima* pods showed significant anti-inflammatory effects when edema-induced rats were fed with pod extract at 400 mg/kg body weight in comparison with the control rats [90]. Pod extract of *Cassia italica* also exhibited anti-inflammatory effects on carragenan-induced paw swelling. The aqueous ethanol (80%) extract also exhibited anti-pyretic activity [91].

The summary of biological activity of compounds of fruit pods associated with health benefits is presented in Table 1 and Table 2. Some potential products that can be suggested from the pod extracts of ethnobotanical plants are those such as anti-inflammatory drugs, antibacterial creams and even dietary supplements which may assist in reducing blood pressure and blood glucose concentration. Although, human clinical trials are a must, positive findings from animal and *in vitro* studies indicate that the benefits of pod extracts should be explored further.

**Table 1:** Bioactive compounds and health benefits of fruit pods.

Pod extract	Bioactive compounds	Health benefits	References
*Acacia nilotica*	Gallocatechin-gallate, methyl gallate, gallic acid, catechin, catechin gallate, galloyl-glucose, digallic acid, ellagic acid, ferulic acid, epicatechin	Reduced fever, antidiarrhea, antioxidant, chemopreventive, antidiabetic, reduced cholesterol, hepatoprotective, antibacterial, antifilarial	[61,62,63,64,65,66]
*Bauhinia purpurea*	Alkaloids, flavonoids, triterpenes, glycosides, steroids	Anti-angiogenic or cancer treatment, hypolipidemic	[73]
*Catalpa bignonoides*	Saponins, sterols, phenols	Anti-inflammatory, antinociceptive	[83]
*Ceratonia siliqua*	Polyphenols: catechin, quercetin, gallic acid, quercetin glycosides, epicatechin gallate, ellagic acid, proanthocyanidins, ellagitannins; Carotenoids: lutein, lycopene, carotene; Dietary fiber	Antioxidant, anxiolytic-sedative effect, chemopreventive, antitumor, antidepressant, antidiarrhea, reduced cholesterol	[41,42,43,44,45,46
*Emblica officinalis*	Hydrolysable tannin: emblicanin, punigluconin, pedunculagin	Antioxidant	[82]
*Garcinia mangostana*	Xanthone: mangostin, gartanin, garcinone, tovophyllin, mangostinone, smeathxanthine, isomangostin, eudraxanthone, methixyxanthone;Polyphenols: afzelechin, epiafzelechin, catechin, epicatechin, gallocatechin, epigallocatechin;Glucose; D-gallacturonic acid, L-rhamnose, D-galactose	Chemopreventive, antibacterial, antifungal, antioxidant, antimalarial, antiallergy, anti-inflammation, anti-obese, reduced cholesterol, enhance immune system	[20,21,22,23,24,25,26,27,28,29,30,31,32,33,34,35]
*Moringa oleifera*	Niaziridin, niazirin, niazimicin, pterygospermin, benzyl isothiocyanate, glucosinolate, carotenoids	Antioxidant, reduced cholesterol, antidiabetic, antiurolithic, hypotensive, anti-inflammatory, hepatoprotective, antibacterial	[47,48,49,50,51,52,53,54,55,56,57,58,59,60]
*Parkia speciosa*	Phenolic, flavonoids	Antioxidant, antidiabetic, antiangiogenic	[74,75,76]
*Prosopis cineraria*	Tripenoids: maslinic acid glucoside; linoleic acid, prosophylline, polyphenols	Antioxidant, antibacterial, antifungal, estrogenic	[70,71,72]
*Samanea saman*	polyphenols: cyanidin, catechin, epicachin, anthocyanin monoglycones, delphinidin, malidin	Antibacterial	[80]
*Sapindus rarak*	Raraoside, rarasaponin, saponins, acyclic sesquiterpene glycosides, hederagenin	Anti-obesity, chemopreventive	[67,68,69]

**Table 2:** Biological activity of Ethnobotanical Fruit Pods.

*Plant pod*	Anti-inflammatory	Anti-inociceptive	Antioxidant	Antibacterial	Antifungal	Reduce fertility	Reduce fever	References
*Albizia julibrissin*			√	√				[78]
*Albizia lebbeck*						√		[79]
*Astragalus hamosus*	√							[86]
*Caesalpinia ferrea*	√							[85]
*Caesalpinia pulcherrima*	√							[87]
*Cassia fistula*				√	√			[81]
*Cassia italica*	√						√	[88]
*Cassia occidentalis*					√			[77]
*Tecoma sambucifolia*	√	√						[84]

## 9. Conclusions

This review has highlighted that most extracts from plant pods (including mangosteen and *M. oleifera*) contain compounds with antioxidant, anti-inflammatory, antibacterial, antifungal and other biological activities*.* The antioxidative properties are mostly related to their high content of polyphenolics such as catechin and gallic acid. In addition, the presence of carotenoids in some pod extracts, such as in carob, can increase the antioxidant capacity, which may suggest a synergystic effect between polyphenols and carotenoid compounds in the pod extract, or with xanthones as in *G. mangostana* pod extract. Potent antibacterial activity against several microbes including *E. coli* and *S. aureus*, as well as antifungal activity towards *C. albicans*, were exhibited by the pod extracts*.* Anti-obesity effects have also been attributed to the extracts from fruit pods of *Sapindus rarak*, *Garcinia mangostana*, *Moringa oleifera* and *Acacia nilotica.* In addition, certain pod extracts can also offer anti-diarrheal effects due to their high dietary fibre content. With these known biological activities, therefore, plant pod extracts can be promoted for usage in pharmaceutical and nutraceutical products in the near future.
